# Revisiting National Life Expectancy: The Effect of Including Prenatal Deaths in Canada

**DOI:** 10.1177/00243639261423447

**Published:** 2026-02-26

**Authors:** Uzair Jamil, Joshua M. Pearce

**Affiliations:** 1 6221 Western University, London, ON, Canada; 2 Ivey School of Business, Western University, London, ON, Canada

**Keywords:** DALY, health policy, life expectancy, life years, pregnancy, terminations

## Abstract

Conventional practices and contemporary international standards for calculating life-years from live birth incorporate a subjective notion of social desirability, wherein only the lifespans of individuals deemed socially acceptable are included in these assessments. These assumptions mask low life-year deaths of socially undesirable humans and obscure results of medical and environmental interventions, thus falsely indicating higher life expectancies for a population. To provide a more comprehensive understanding of national health trends, this study introduces a methodology that incorporates individuals considered socially undesirable into life expectancy estimates. A case study is presented for Canada to evaluate the national life expectancy and planned prenatal deaths from 1970 to 2020. When accounting for pregnancy terminations, the actual life expectancy is approximately 65.8 years—16.7 years less than the 2020 estimate of 82.5 years, reflecting a 20% decrease. The maximum difference of 20.5 years occurs between the official estimate and the revised life expectancy in 2002. From the results it is evident that published Canadian life expectancies are incorrect. A comprehensive global investigation is required, along with a refinement in life expectancy calculations that avoids bias by accounting for life expectancy of both socially desirable and undesirable humans.

## Introduction

Following conventional practices that focus only on the lifetimes of socially desirable humans, both globally and at the national scale, human life expectancies have been increasing (Dattani et al. 2023; Government of Canada 2013). In 2022, however, Canada experienced a decline in life expectancy for the third consecutive year, dropping to 81.3 years (Government of Canada 2023). This reduction is due to unintentional injuries, suicides, homicides, and the impacts of COVID-19 (Government of Canada 2023). The standard model of life expectancy attributes improved life expectancy to several factors. These include advancements in childhood immunization, the discovery of penicillin and insulin, and new strategies in illness prevention and health promotion (Government of Canada 2013). Similarly, reduction in mortality rates from other diseases, such as heart diseases, has also enhanced life expectancy (Government of Canada 2013). The improvement is also in part a result of healthy dietary patterns and affordable food (Barkat et al. 2024; Fadnes et al. 2023).

Conventional practices and contemporary international standards that perform life expectancy estimations incorporate a tacit notion of social desirability. This means that only the lifespans of individuals deemed socially acceptable are included in these assessments (Arias 2015; CDC 2019; Government of Canada 2019). This assumption extends to subsequent analyses, such as the disability-adjusted life year (DALY), which widely inform health policy decisions (Murray and Lopez 1997*a*, 1997*b*). This assumption, however, could potentially obscure the extent of life-years lost due to socioeconomic or other factors. Subsequently, this leads to inaccurate life expectancy estimates within a given population. For example, when an individual diagnosed with a medical condition that significantly reduces life expectancy is terminated before birth due to perceived social undesirability, they are not included in the national life expectancy calculations. As a result, omitting purposeful prenatal deaths may hide the influence of key medical and environmental factors, as well as critical intervention areas and health policies that would otherwise affect population longevity. To provide a more comprehensive understanding of national health trends, this study introduces a methodology that incorporates individuals considered socially undesirable into life expectancy estimates. A case study is presented for Canada, and the findings are discussed in the context of improving health policy at national and international levels.

## Methods

A 51-year period (1970–2020) is investigated using the historical pregnancy termination statistics for Canada from Johnston's Archive along with the birth counts (Johnston 2024). Calculations were stopped at 2020 to avoid complexities of COVID-19 and started in 1970, as although the data from the Johnston's Archive dates back to 1921 for live births, the data for socially undesirable terminations is only collected from 1970 onwards (Johnston 2024). To include pregnancy terminations of socially undesirable (*su*) humans into life expectancies, the following equation is used for the corrected life expectancy (*LE_corrected,su_*):

(1) $$LE_{corrected,su}(years)=L\;\times \left(\frac{b_{t}}{b_{t}+u_{t}}\right)$$

where *L* is the conventional standard life expectancy at birth in years, *b_t_* is the number of socially desirable births/year in a given year *t*, and *u_t_* is the number of terminations of undesired pregnancies in year *t*.

When accounting for fetal deaths, defined as the spontaneous intrauterine demise of a fetus at any stage of pregnancy—commonly occurring later in pregnancy (e.g., at 20 weeks of gestation or more, or 28 weeks or more)—the equation is adjusted as follows:

(2) $$LE_{corrected,su-f}(years)=L\;\times \left(\frac{b_{t}}{b_{t}+u_{t}+f_{t}}\right)$$

where *f_t_* is the number of fetal deaths in a given year *t*.

It is pertinent to clarify that these estimates do not differentiate pregnancy terminations due to medical conditions or otherwise. All abortions/fetal deaths are benchmarked against the national life expectancy values. Furthermore, certain medical conditions, such as Down syndrome, significantly reduce the lifespan of individuals (O'Leary et al. 2018). Not accounting for their actual life years may skew the projections of the corrected estimates presented in the current study.

The Canadian case study data was further stratified to calculate life expectancy for each province, taking the year 2016 as a sample case. Data on terminations of undesired pregnancies was sourced from the Canadian Institute for Health Information (Canadian Institute for Health Information 2016), while the provincial average life expectancy data was obtained from Statistics Canada (2014–2016) (Government of Canada 2017). Moreover, the data on live births for each province was acquired from Statista (2015/2016) (Statista 2016). Although the same general formula (equation (1)) was applied, it was customized for each province. The equation (1) can be expressed individually for each province as follows:

(3) $$LE_{corrected,su,province}(years)=L_{\;province}\;\times \left(\frac{b_{t,province}}{b_{t,province}+u_{t,province}}\right)$$

## Results and Discussion

**Figure 1.**
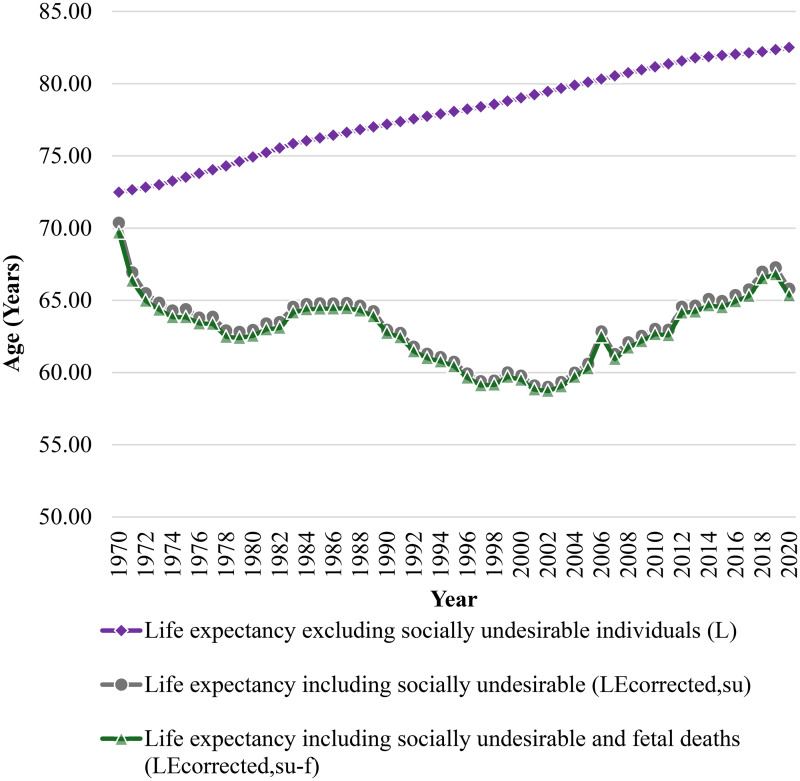
Life expectancies from 1970 to 2020 as a function of years with and without social undesirable deaths included.

Traditional calculated life expectancy is compared to the life expectancy of Canada including terminations of undesired pregnancies in Figure 1. Traditional life expectancy measured only for socially desirable humans in Canada has increased from 72.5 to 82.5 years from 1970 to 2020 (purple diamonds in Figure 1 starting from 1970). This is a 10-year increase in life expectancy in 70 years and follows a monotonic and even linear increase.

After including the count of pregnancy terminations of the socially undesirable, however, it is clear from Figure 1 that the life expectancy does not follow a monotonic traditional increasing trend. The primary change in the corrected life expectancy shown in Figure 1 is due to the legalization of prenatal termination in Canada in 1988 (Long, Foot and McIntosh 2022). Life expectancy including the count of pregnancy terminations (gray circles in Figure 1) is well below the traditional life expectancy for all years evaluated. Similarly, incorporating fetal deaths into the analysis results in a further reduction in life expectancy, though the decrease is marginal (green triangles in Figure 1). The proportion of fetal deaths compared to reported abortions was approximately 3.9% on average during the study period.

The difference between the simplistic or traditional life expectancy, *L*, and the complex *LE_corrected,su_/LE_corrected,su−f_* related to socially undesirable people gradually increases over time. In 1970, the difference for LE including socially unwanted individuals was 2.11 years, while in 2020, it reached 16.71 years. Incorporating fetal deaths, the numbers are slightly higher with values reaching 2.79 and 17.15 years, which suggests an expanding gap in outcomes for populations affected by these events. In 2020, the *LE_corrected,su_* and *LE_corrected,su−f_* were 65.81 and 65.36, respectively, compared to the traditional LE of 82.52 years. The number of reported pregnancy terminations increased significantly over time. The number reached 115,556 in 2007 and increases to 118,193 if fetal deaths are included. Terminations of undesirable pregnancies represented 31.41% of the total live births considering 367,864 live births were recorded in the same year. This proportion did not account for fetal deaths, which will augment the value further. When viewed in the context of Canada's total population of 32,852,800 in 2007 (Statistics Canada 2007*a*), unwanted pregnancy terminations represented around 0.36% of the population that year.

The highest difference between the traditional life expectancy and the life expectancy incorporating socially undesirable individuals occurs in 2002, with a difference of 20.45 years (20.72 years including fetal demises). This was derived from the official national life expectancy reported by Macrotrends, 79.47 years (Macrotrends 2024), and corrected life expectancy estimated using equation (1), 59.01 years. The corrected life expectancy includes 105,669 reported terminations and 308,617 live births for that year—data sourced from Johnston's archive (Johnston 2024). This demonstrates the implications of including terminated unwanted pregnancies in life expectancy calculations. The statistics are affected, although the postnatal survival values stay the same.

The lowest difference is observed in 1970, with a difference of 2.11 years. In this year, the overall life expectancy is 72.49, while the *LE_corrected,su_* is 70.38 years. Incorporating socially nondesirable individuals into calculations of life expectancy results in a reduction of approximately 14.74 years on average through the period of 1970 to 2020; whereas including fetal deaths in the analysis results in a reduction of life expectancy by 15.11 years.

**Figure 2.**
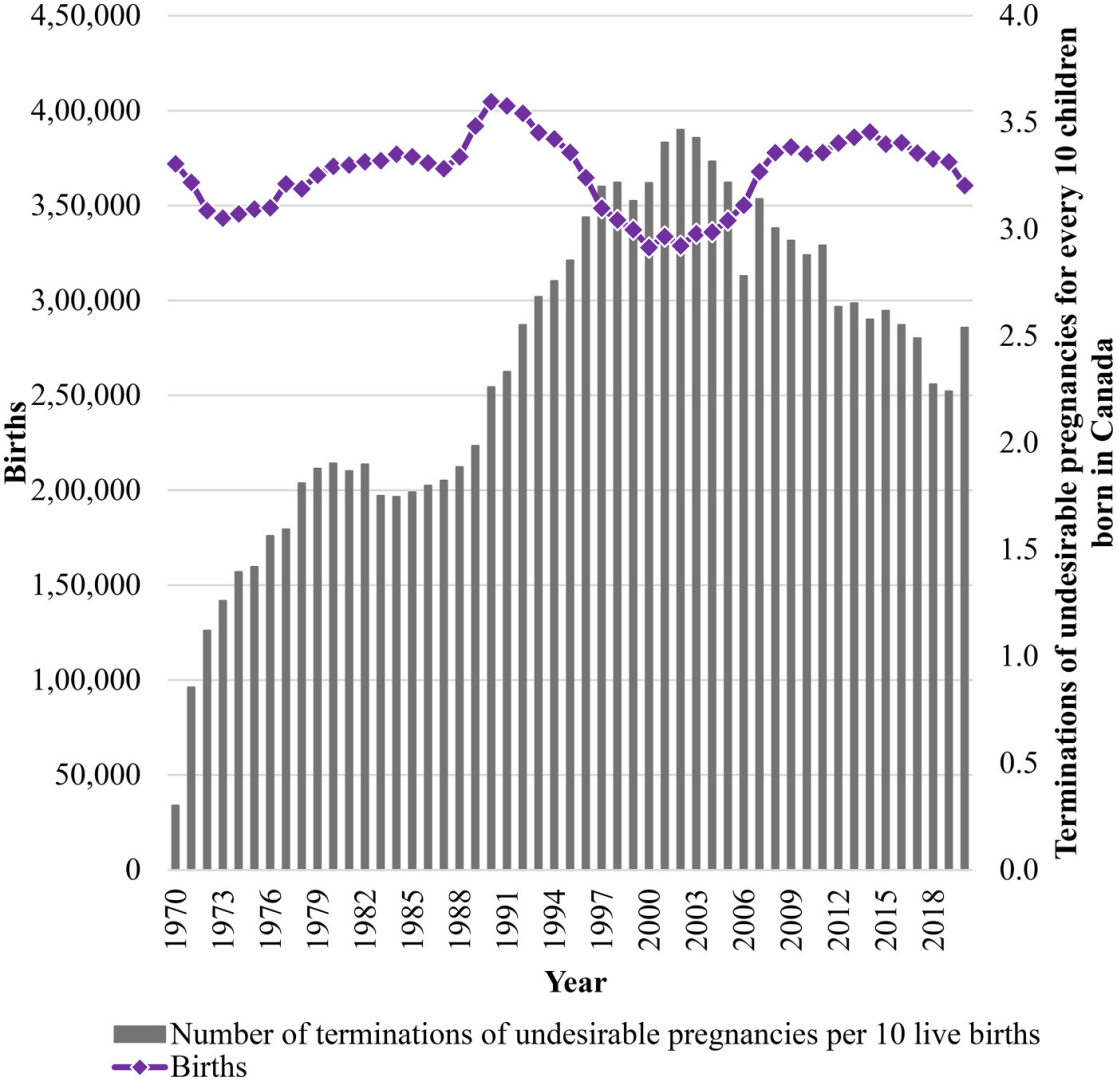
Number of terminations of undesired pregnancies compared to live births in Canada.

The data in Figure 2 shows a steady rise in the number of terminations of undesired pregnancies per 10 live births from 0.3 in 1970 to a peak of 3.5 in 2002. This upward trend is quite prominent from the late 1980s through the early 2000s. After 2002, a gradual decline is observed. The ratio decreases to 2.2 in 2019 before witnessing a slight increase to 2.5 in 2020. These changes reflect fluctuating trends in terminations of undesirable pregnancies over time. There has been an increase in live births between 2000 and 2010, which combined with the number of terminations is associated with the decline in the ratio of terminations to live births. This is an explanation of the aforementioned variation. The increase in the number of births from 2000 onwards has been linked to the desire to have a child during the first year of the new millennium (Statistics Canada 2005). Additionally, a significant rise in the number of marriages during this period may have contributed to this trend (Statistics Canada 2007*b*). The rise in birth rates during this time (1.5% more in 2005 than the preceding year) is primarily attributed to two key factors: an increase in the population of women of reproductive age and a potential rise in fertility rates (1.54 children per woman) (Statistics Canada 2005).

**Figure 3.**
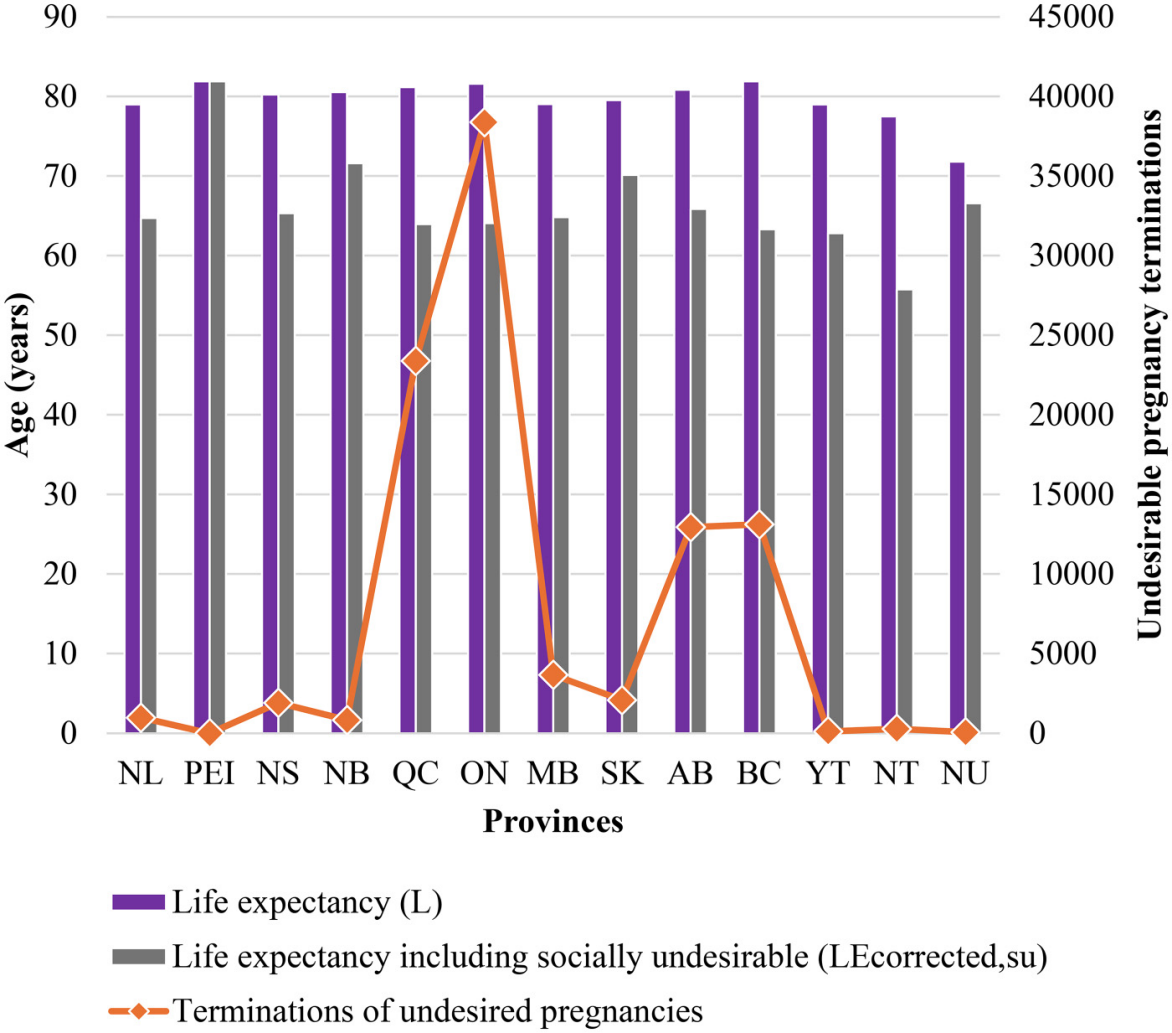
Provincial life expectancies and terminations of undesired pregnancies.

A more granular approach to the analysis of individual provinces provides more insight. Provinces with higher numbers of terminations of undesired pregnancies show a significant decline in life expectancy when this factor is included. In provinces with high live birth rates, such as Ontario and Quebec, the contrast between life expectancy with and without considering socially undesirable factors is especially striking. Both provinces experience significant reductions in life expectancy when factoring in pregnancy terminations, despite having the highest number of live births (140,582 and 86,947, respectively). For instance, Ontario, with 38,383 terminations, showed a recalculated life expectancy of 64.08 years compared to the traditional estimate of 81.58 years, when terminations of undesirable pregnancies are taken into account. In Quebec, with 23,393 terminations of undesirable pregnancies, life expectancy calculations reflect a drop from 81.16 to 63.95 years. Moreover, corrected life expectancies were estimated to be higher in provinces of Saskatchewan and New Brunswick (70.13 and 71.62 years). This was primarily because of the relatively lower termination rates compared to Quebec and Ontario. Conversely, in Prince Edward Island, where no terminations of undesired pregnancies were recorded, life expectancy remains high at 81.9 years. The estimates with the revised formulae presented in the study record no decline. In smaller territories like Yukon and Northwest Territories, the number of terminations was relatively low (116 and 277, respectively). Despite this, the impact on life expectancy was considerable. The revised estimate in Yukon was 62.81 years reduced from 79 and 55.75 in Northwest Territories from 77.5, mainly because of the relatively low populations (Figure 3).

**Figure 4.**
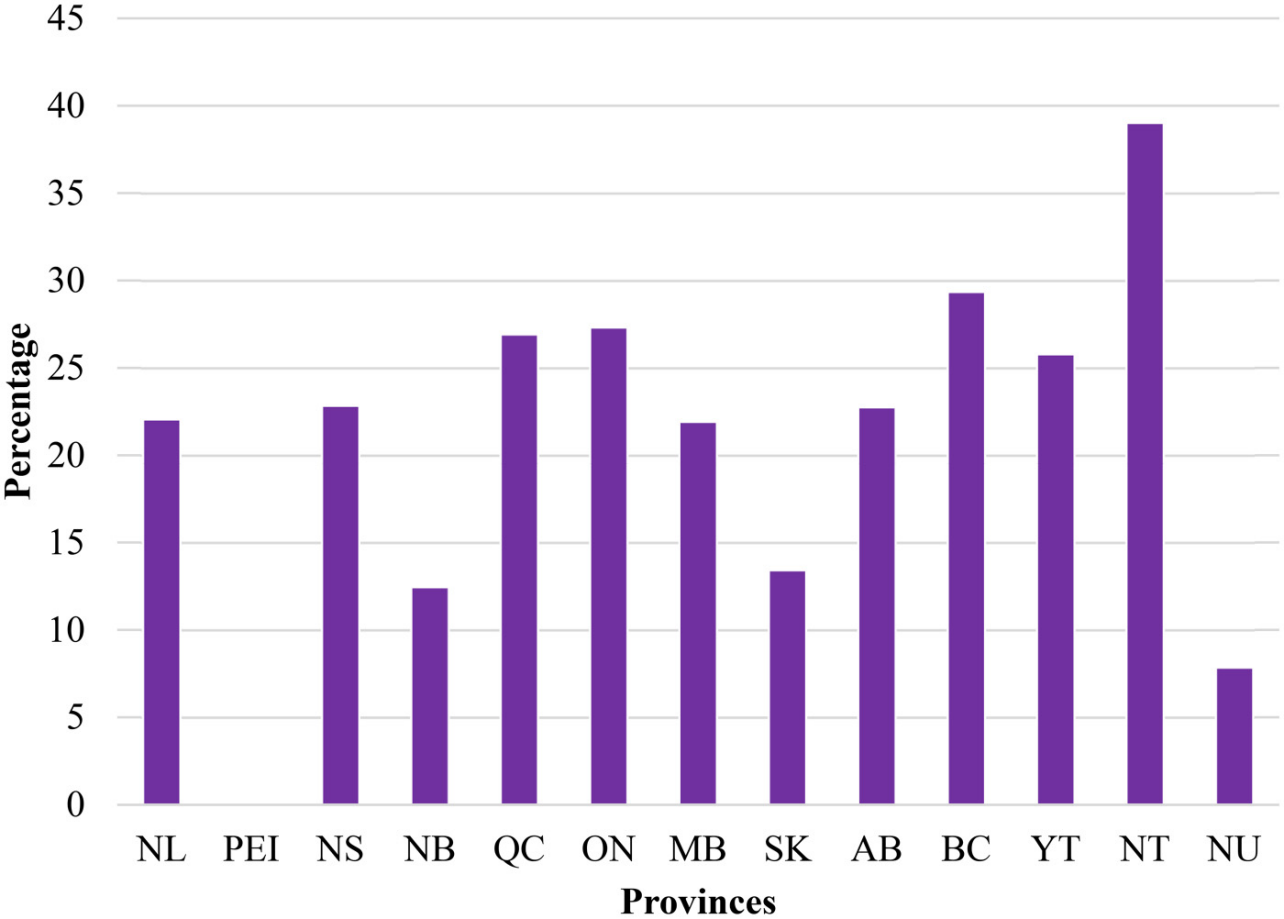
Terminations of undesired pregnancies as a percentage of live births for each province in Canada.

To clarify this point, Figure 4 shows a notable variation in terminations of socially undesired pregnancies as a percentage of live births across the different provinces and territories in Canada. British Columbia registers a high rate of 29%, while the Northwest Territories tops the list at 39%. In contrast, Prince Edward Island reports no terminations, while Nunavut has one of the lowest rates at 7.8%. Quebec and Ontario, provinces with the largest populations in Canada, demonstrate substantial rates, 26.9% and 27.3%, respectively. These numbers are despite the fact that the provinces registered the highest number of live births. These findings suggest significant regional differences in terminations of socially undesired pregnancies as a percentage of live births. The reason could be factors such as access to healthcare services, cultural attitudes, or provincial policies on pregnancy and reproductive health. Future work is needed to determine what these differences are and how the real-life expectancy can be improved for the weaker performing provinces. How, for example, can the corrected life expectancy of the Northwest Territories be improved to that of their neighbors in Nunavut?

Research has indicated that economic factors play a significant role in decisions regarding pregnancy termination, regardless of marital status (Adelson, Frommer and Weisberg 1995). Young married women may choose to terminate a pregnancy for economic convenience, until the family becomes financially stable (Ford and Nault 1996; Wadhera and Millar 1991). This trend also correlates with declining fertility rates in this group (Ford and Nault 1996; Wadhera and Millar 1991). Career and educational goals also appear to play a crucial role in abortions (King, Myers and Byrne 1992; Medoff 1988). Studies have indicated that women whose careers would either be more inconvenienced or with stronger economic positions are more likely to opt for pregnancy termination (King, Myers and Byrne 1992; Medoff 1988). It has been suggested that unmarried women may face greater economic consequences from nonmarital pregnancies (Trent and Powell-Griner 1991). Such pregnancies can carry a higher level of social disgrace and embarrassment as well (Trent and Powell-Griner 1991; Wiegers and Chunn 2015). Additionally, regional pregnancy terminations have been found to correlate with factors such as per capita disposable income and local unemployment levels (Rothstein 1992). As income rises by approximately $2,000, the demand for abortion increases by 4% (Rothstein 1992). This trend can be largely attributed to the increased value of women's time and a corresponding decline in the demand for children (Rothstein 1992). Conversely, when the unemployment rate rises by 1.4%, the abortion rate decreases by 1.39% (Rothstein 1992). This decline may be explained by either a decrease in the value of women's time or by individuals becoming more cautious about contraceptive use (Rothstein 1992).

This study highlights the urgent need for more thorough and accurate data collection to gain a better understanding of life expectancy in Canada. Currently available data is often incomplete or misleading—for instance, no data on ethnicity or race is available for individuals opting termination in Canada, unlike the United States, where data is available on pregnancy terminations for population groups such as Hispanic, African American, and Caucasian, and detailed demographic comparisons can thus be made (Hollman and Pearce 2023). The approach introduced here can also be applied in other nations to refine estimates of life expectancy, though cross-country comparisons are complicated by varying and evolving legal frameworks. Prior studies have also documented the efficacy of determining postconception life expectancy to improve the survival chances of humans (Meidl 2009).

The influence of diverse legal and social practices on life expectancy estimates must not be overlooked, as they can lead to discrepancies in standard estimations. For example, if a country determines that those with certain genetic abnormalities that reduce lifetime are deemed socially undesirable and terminated before birth, the country's life expectancy will increase calculated in the traditional way, even though no one is actually living longer. This phenomenon appears to already be in practice. For instance, individuals with Down syndrome, who typically have a life expectancy of around 60 years, contrast sharply with the broader life expectancy estimate of 82.52 years for the general Canadian population. The percent of Down syndrome Canadians terminated before birth is unknown, but various sources show that in the United States for instance, 60% to 90% of children diagnosed with Down syndrome are terminated (Natoli et al. 2012). Estimates suggest that without selective abortion, 80% more babies with Down syndrome would be born each year, and the Down syndrome population would grow by an additional 217,000 people over the next 50 years—an increase comparable to the current Down syndrome population size in the United States (United States Joint Economic Committee 2022). Further research is necessary to apply the corrections outlined in this study to Canada life expectancy estimates and to compare these results with other countries, where the termination rate for Down syndrome pregnancies varies considerably (e.g., 77% in France, 98% in Denmark, and 100% in Iceland) (Tzoganakis 2021).

Historically, however, life expectancy has been calculated based on live births and age at death. The methodology is favored because of its simplicity and widespread availability of data used in its calculation. Adopting this approach makes it convenient to record changes over time. Moreover, it also facilitates comparisons across populations, regardless of differences in policies regarding pregnancy terminations. As shown in this study, the focus on simplicity and historical comparability has come at the expense of capturing information that could improve health outcomes for the populations under study. The corrected life expectancy indicator (*LE_corrected,su_*) serves as an alternative approach to provide a more comprehensive assessment of population health. Unlike traditional life expectancy measures, the novel technique suggested in the current study does not only account for live births. The proposed indicator acknowledges a broader range of pregnancy outcomes, offering a perspective that aligns more closely with the complexities of health and wellbeing in the population.

The findings of this study can significantly contribute to reforming health regulations. This is especially relevant for public policies aimed at improving health conditions that contribute to socially undesirable outcomes, such as congenital anomalies, which ultimately result in abortions (Heaney, Tomlinson and Aventin 2022). The indicator could also contribute in the development and implementation of public health interventions. Such interventions may take the form of improved maternal health services or targeted education campaigns. Ample evidence exists which demonstrates that initiatives like folic acid supplementation have effectively reduced the prevalence of certain conditions (Scholl and Johnson 2000) that might otherwise lead to terminations of socially undesirable pregnancies. A study in the United States analyzed 227 abortion cases for “screened for” conditions (Grossman and Chasen 2020). Overall, 93% (210) of those cases were linked to karyotype abnormalities (Grossman and Chasen 2020). These were followed by 5% (12) cases due to single-gene disorders (Grossman and Chasen 2020). Lastly, 2% (5) cases were due to microarray abnormalities and 5% (12) were due to single-gene disorders (Grossman and Chasen 2020). Research conducted in the United Kingdom identified various factors resulting in termination of pregnancies, These included chromosomal anomalies, such as trisomy 21 (Down syndrome), as well as structural defects involving the nervous and musculoskeletal systems (Royal College of Obstetricians and Gynaecologists 2010). Using the broader characterization of life expectancy would add policy support for preventing anomalies that have been resulting in uncounted life expectancy due to terminations.

One limitation of this study is that the proposed methodology is more complex and less easily implemented than traditional methods. This becomes especially relevant when calculations are performed in countries where required data is not systematically collected. Further research is necessary to apply this method to other countries and to aggregate the findings for a global perspective. In addition, the study did not exclude pregnancy terminations performed to protect the health of the mother. As only 0.3% of abortions occur after 20 weeks of gestation, with the majority arising from severe fetal or maternal health complications (Joyce Arthur 2006), approximately half of these cases can be attributed primarily to maternal health concerns. The impact of this proportion on the study's findings and the calculated life expectancy values, however, was negligible. Moreover, the study is limited by the year range that it covered.

Canada lacks data regarding the reasons for pregnancy termination (Abortion Rights Coalition of Canada 2020). This adds another limitation to the current study. It prevents distinctions between pregnancies that were terminated due to the social undesirability or medical conditions, such as familial hypercholesterolemia or trisomy 21 (Down syndrome), or other factors. Individuals suffering from these illnesses die earlier than the normal population (O'Leary et al. 2018). Including the actual lifespan for these terminations in the proposed life expectancy calculation would result in a higher value of corrected life expectancies (*LE__(corrected,su)_* or *LE__(corrected,su−f)_*). Therefore, this will lower the impact of abortions on revised calculations. Future research could ascertain the reasons of pregnancy terminations and quantify their implications on national life expectancy assessments.

While it is well-established that improvements in public health (Cutler and Miller 2005), reductions in infectious diseases (Crimmins 2015), and the use of antibiotics (Jayachandran, Lleras-Muney and Smith 2010) have contributed to increased life expectancy, this study demonstrates that the traditional method of calculating life expectancy may overstate the true magnitude of these gains by excluding prenatal deaths from consideration. The collection of comprehensive, accurate data—including all deaths—is imperative. The global medical community can then use this information to develop informed policies that improve health metrics. Ultimately, better medical policy, informed by more accurate life expectancy estimates, could lead to improved prenatal care and enhanced mental health outcomes.

## Conclusions

Life expectancy calculations in Canada, when accounting for pregnancy terminations, reveal significant disparities. The maximum difference between official estimates and reality is almost 20.45 years. Current Canadian life expectancy figures are skewed by the exclusion of individuals who are not factored into these calculations due to being classified as socially undesirable. When pregnancy terminations are included, the actual life expectancy is approximately 65.81—16.71 years lower than the 2020 estimate of 82.52 years, which is 20% less. These findings highlight the necessity for a redefinition of life expectancy metrics in Canada, and globally, to eliminate the bias introduced by excluding prenatal deaths. Accurate, transparent, and comprehensive data on all pregnancies is critical for shaping effective health policies. Such measures then guide both national and international regulations aimed at improving public health.
